# Effects of sediment influx on the settlement and survival of canopy-forming macrophytes

**DOI:** 10.1038/srep18677

**Published:** 2016-01-04

**Authors:** Hayato Watanabe, Miku Ito, Akira Matsumoto, Hisayuki Arakawa

**Affiliations:** 1 Corporate Planning Department, Iwate Prefectural Government, 1-1 Youkacho, Kuji, Iwate 028-0064, Japan; 2 Tokyo University of Marine Science and Technology, 4-5-7 Konan, Minato-ku, Tokyo 108-8477, Japan

## Abstract

Kelp forests on coastal rocky shores are negatively impacted by sudden sediment loads that can occur with storms and floods. Using laboratory experiments, we studied the effects of sediment deposition on the survival of the large brown alga *Eisenia bicyclis* juveniles (zoospores and gametophytes) to quantify the potential impacts of particulate matter on kelp forests. The zoospore adhesion rate and the gametophyte survival and growth rates all declined markedly with increasing sediment load, particularly with smaller particle diameter. Using experimental results, we derived an equation to calculate the rate of initial kelp depletion with sediment load based on the quantity and size distribution of sediment particles. The equation enabled the estimation of *E. bicyclis* depletion rates in the field by measurement of particle quantity and diameter distribution of sediments on the reef substrate.

Kelp forests are marine communities established by brown macrophytes such as *Eisenia bicyclis* and *Ecklonia cava* that serve as valuable feeding and nursery grounds for a diverse range of coastal life^1^,^2^. However, kelp forests are affected by various factors including water temperature^3^,^4^, nutrients^3^,^5^,^6^, the strong currents associated with storms and waves^2^, grazing by herbivorous organisms^7^,^8^,^9^ and competition from other algae^2^,^10^, which result in a cycle of expansion and reduction of the forest area. Changes in turbidity and seabed sediments also affect kelp forests. Pulsed sediment loads occurring from natural phenomena, e.g., river floods^11^,^12^, cliff erosion and runoff^13^, resuspension after storms^2^,^14^,^15^ and artificial processes including construction and solid waste disposal^16^,^17^,^18^ can inflict serious harm on these macrophyte species.

Increased seawater turbidity and/or sediments can impact marine kelp communities either by indirectly reducing sunlight or by the direct physical influence of the particulate matter. Studies have examined the effects of reduced sunlight on seaweed beds inhabiting coastal areas^19^,^20^,^21^. The direct impacts of particulate matter have been studied on several species inhabiting temperate reefs^22^,^23^,^24^,^25^. For example, sediments may directly influence the canopy-forming macrophyte *Macrocystis* by inhibiting sporophyte adhesion^22^. Growth of *Undaria pinnatifida*^26^, another brown macrophyte species, is also inhibited by sediment particles. Studies have shown that the rate of brown macrophyte zoospore adhesion is greatly reduced by the accumulation of particulate matter on the substrate, and sediment settlement atop zoospores and gametophytes of these species reduces growth and survival^27^,^28^,^29^. However, these studies examined one particle size in the laboratory and did not encompass the wide range of particle sizes occurring in the field. In contrast, field experiments on turf-forming algae have shown that increasing sediment quantities promote turf algae while inhibiting canopy-forming macrophytes^23^,^24^,^25^.

Using laboratory experiments, we determined the effects of different sizes of sediment particles on zoospore adhesion and gametophyte growth and survival of the canopy-forming macrophyte *E. bicyclis*. We used this data to develop an equation estimating the rate of initial *E. bicyclis* depletion under sediment loads that more accurately estimate the effects of reef sediment deposition on kelp forests.

## Results

### Effects of sediment particles on *E. bicyclis* zoospore adhesion

**Figure 1:**
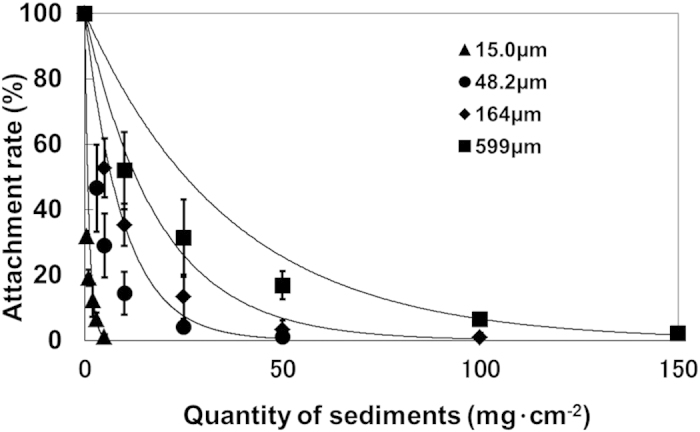
Relationship between sediment quantity and zoospore attachment rate for each sediment particle size.

Relative to the control, the *E. bicyclis* zoospore adhesion rates in the presence of 15.0-μm particles were 31.8 ± 1.6%, 19.2 ± 2.5% and 1.2 ± 0.6% under the 1, 2 and 5 mg sediment cm^−2^, respectively. Thus, adhesion declined as a logarithmic function of increasing particle quantity (Fig. 1). The adhesion rate in treatments with 48.2, 164 and 599-μm particles also declined logarithmically with increasing sediment quantity. With the 10 mg cm^−2^ sediment, the adhesion rates were 14.4 ± 6.6%, 35.4 ± 11.9% and 51.9 ± 9.0% in the 48.2, 164 and 599-μm particle size treatments, respectively, indicating that smaller particles have greater negative effects for a given volume of sediment.

Based on the exponential approximation of the adhesion rate in the absence of sediment (defined as 100%), the relationship between the *E. bicyclis* zoospore adhesion rate and sediment quantity for each particle diameter was defined as:


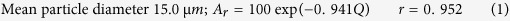



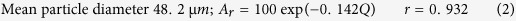



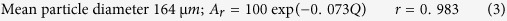



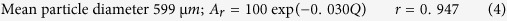


where, *A*_*r*_ and *Q* denote the relative *E. bicyclis* zoospore adhesion rate (%) and the quantity of sediment particles (mg cm^−2^), respectively.

### Effects of sediment particles on gametophyte growth and survival

**Figure 2:**
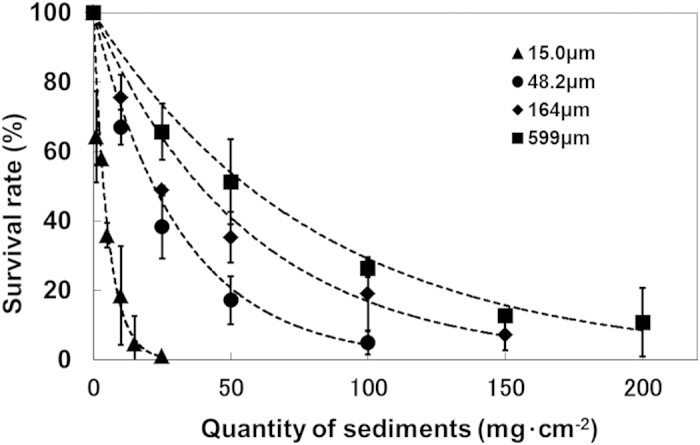
Relationship between sediment quantity and the gametophyte survival rate for each sediment particle size.

Survival of *E. bicyclis* gametophytes declined markedly as sediment quantity increased (Fig. 2). Relative to the controls with no sediment, gametophyte survival rates under 15.0-μm diameter particles of kaolinite were 64.2 ± 13.2%, 22.3 ± 18.7% and 0% in the 1, 10 and 25 mg cm^−2^ treatments, respectively. Survival rates under 48.2, 164 and 599-μm particles also declined as a logarithmic function of particle quantity. Additionally, smaller particle sizes had stronger negative effects on survival. With 25 mg cm^−2^ sediment, the survival rates were 38.3 ± 9.2%, 48.8 ± 1.4% and 65.7 ± 8.1% in the 48.2, 164 and 599-μm particle size treatments, respectively.

Exponential approximation of the relative gametophyte survival rates for different particle diameters gave the following relationships:


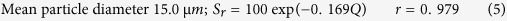



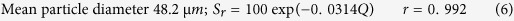



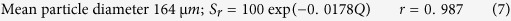



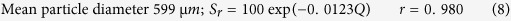


where, *S*_*r*_ and *Q* denote the relative *E. bicyclis* gametophyte survival rate (%) and the quantity of sediment particles (mg cm^−2^), respectively.

The total length of *E. bicyclis* gametophytes on day 12 is shown for each particle size in Tables 1 and 2. In the absence of sediment, mean gametophyte length was 28.3 ± 2.0 μm on day 6, and 144.9 ± 3.9 μm for male and 164.8 ± 3.9 μm for female gametophytes on day 12. Analysis of covariance (ANCOVA) revealed that the negative effects of sediment on gametophyte growth increased both with increasing sediment quantity and decreasing particle size (*P* < 0.001). Additionally, gametophyte length differed significantly between males and females (*P* < 0.001). However, there were no interactions between either, particle size and sex (*P* = 0.900) or sediment quantity and sex (*P* = 0.274), indicating no difference in the effects of sediment on gametophyte growth between males and females.

### Estimation of field rates of *E. bicyclis* depletion under varying sediment loads

The present study revealed the relationships between the *E. bicyclis* zoospore adhesion rate and the quantity of sediments with different particle diameters [equations (1, 2, 3, 4)], as well as the relationships between the *E. bicyclis* gametophyte survival rate and the quantity of sediments with different particle diameters [equations (5, 6, 7, 8)]. Here, we used these equations to estimate the expected fields rates of *E. bicyclis* zoospore adhesion and gametophyte survival based on the quantity and diameter of sediment particles deposited on the substrate.

**Figure 3:**
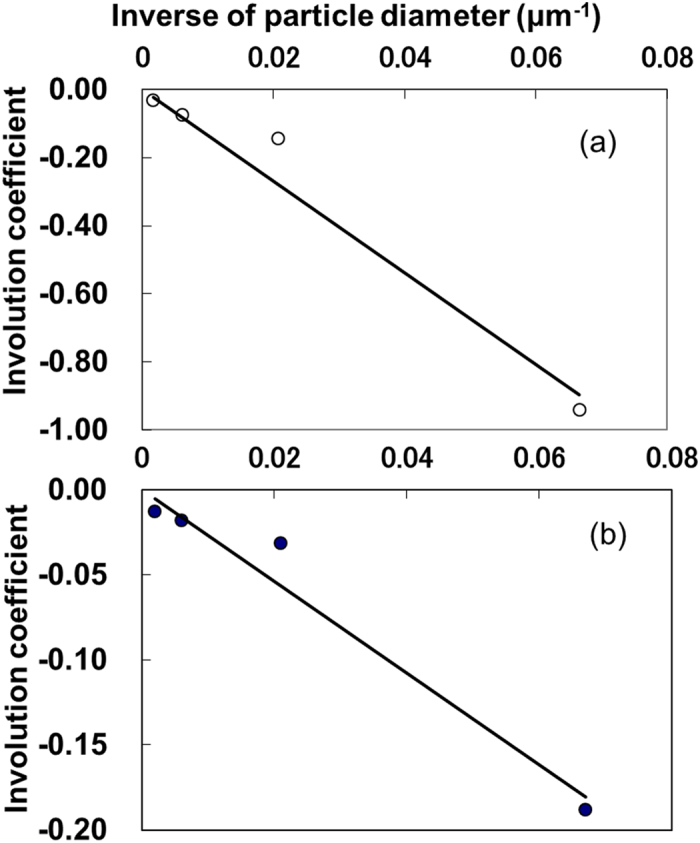
Relationship between the reciprocal of particle diameter and the involution coefficient of (**a**) the adhesion rate as determined by equations 1, 2, 3, 4, 5 and (**b**) the survival rate as determined by equations 5, 6, 7, 8.

Equations (1, 2, 3, 4) and (5, 6, 7, 8) describe the effects of uniformly sized particles. Arakawa *et al.*^30^ showed that the inhibitory effect of sediment on *Gelidium elegans* spore adhesion was greater under a range of particle sizes than under those of a uniform diameter, even when both sets of particles had an identical mean diameter. Because particulate matter on the seabed is composed of particles of varying diameter, equations estimating the effects of a wide range of particle diameters are needed. Based on the relationship between the *E. bicyclis* zoospore adhesion rate and sediment quantity, the relationship between the inverse of particle diameter (*x* = *1*/*D*) and the coefficient (*y*) in equations (1, 2, 3, 4) was determined to be (Fig. 3a):


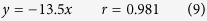


Combining equations (1, 2, 3, 4) with equation (9), the zoospore adhesion rate (%), *A*_*r*_, can be expressed by the mean particle diameter *D* (μm) and the total quantity *Q* (mg cm^−2^) of sediment:


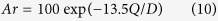


The *E. bicyclis* zoospore adhesion rate can thus be calculated when the mean particle diameter and quantity of sediment are known.

Similarly, the effect of sediment particles on the *E. bicyclis* gametophyte survival rate can be calculated using the inverse of particle diameter (*x* = *1*/*D*) and the coefficients (*y*) from equations (5, 6, 7, 8) (Fig. 3b):


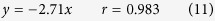


Combining equations (5, 6, 7, 8) with equation (11), the gametophyte survival rate (%), *S*_*r*_, can also be expressed as a function of *D* (μm) and *Q* (mg cm^−2^), which allows its calculation when *D* and *Q* are known:


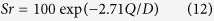


Consequently, the overall effect of sediment particles on the initial *E. bicyclis* depletion rate (%) (zoospores and gametophyte survival) can be derived from the mathematical product of equations (10) and (12):


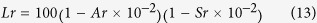


where, *L*_*r*_, *A*_*r*_ and *S*_*r*_ denote the initial depletion rate, the zoospore adhesion rate and the gametophyte survival rate, respectively. *L*_*r*_ represents the rates of detachment and death of zoospores and gametophytes caused by sediment particles. By inserting equations (10) and (12) into equation (13), an equation can be derived that expresses the effect of the quantity (*Q*) and particle diameter (*D*) of sediments on the early stages of *E. bicyclis* (*L*_*r*_):


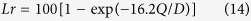


Because sediments on the seabed have a wide range of diameters, and the use of mean particle diameter underestimates the effects of sediment^30^, we applied the technique of Arakawa *et al.*^30^ to solve this problem. When the range of particle diameters in equation (14) are divided into *n* groupings, the initial *E. bicyclis* depletion rate (*Lr*) can be expressed as the mathematical product of the effects of particles in each diameter grouping:


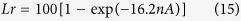



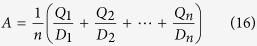


Thus, initial *E. bicyclis* depletion can be estimated from the quantity and size distribution of sediment particles on the substrate.

## Discussion

Some of the factors that affect kelp forest dynamics include temperature and nutrient stress, strong surge and flow during storms, sand and sediment scour, grazing and competition from other algae^2^. Canopy-forming communities are depleted by unusually high water temperatures and low nutrient concentrations^8^. Dayton *et al.* also reported the destruction of a *Macrocystis* kelp community by strong winter storms^2^. There are many reports of biological disturbances like grazing and competition in kelp communities^7^,^8^,^9^. That is, these physical and biological factors cause the depletion and expansion of canopy-forming communities, interfering with the very complex dynamics of the communities. Fluctuations in suspended seawater particles and seabed sediment volumes also affect kelp communities^31^. In coastal rocky areas, natural phenomena such as river floods^11^,^12^, accumulation after storms^14^,^15^ and sediment scour by strong water currents create sediment loads^32^,^33^,^34^.

The sediment loads from natural phenomena on rocky reefs have a considerable influence on the early life-stages of kelp forest species. In this paper we focused on the effects of sediments on the early life-stages of canopy-forming algae. Our study found that increasing sediment deposition on the substrate inhibited *E. bicyclis* zoospore adhesion, and the inhibitory effect was more prominent as particle diameter decreased. Larger sediment quantities also reduced *E. bicyclis* gametophyte growth and survival.

Inhibition of the larval and juvenile stages by sediments has been reported in many algal species^21^,^22^,^23^,^27^,^28^,^35^,^36^,^37^,^38^,^39^,^40^,^41^,^42^,^43^,^44^. However, there are few reports on the differences in negative effects on canopy-forming algae by different size sediment particles. According to reports, smaller sediment particles have larger negative effects on early stage canopy-forming alga^30^,^45^,^46^.

Arakawa *et al.*^30^ also investigated the adhesion of *G. elegans* spores on substrates upon which sediment particles of varying sizes were deposited in different quantities. Similar to the current study, they showed that less spores adhered as the quantity of particles of any size increased. Additionally, the inhibitory effect on adhesion increased with decreasing particle size. However, the effects were stronger on *E. bicyclis* than on *G. elegans* for a given particle size. Arakawa *et al.*^30^ also found that kaolinite particles inhibited *G. elegans* spore adhesion at loads of 30 mg cm^−2^. This result, combined with those of previous studies^28^,^29^, indicates that a given quantity of sediment particles also inhibits substrate adhesion in *E. bicyclis*, *E. cava* and *U. pinnatifida* zoospores, but is less inhibitory to *G. elegans.* This effect is likely attributable to size differences, where *E. bicyclis*, *E. cava* and *U. pinnatifida* zoospores are ~5 μm in diameter^27^, those of *G. elegans* are 30 μm^47^. Thus, comparison between our results on *E. bicyclis* and those on *G. elegans* by Arakawa *et al.*^30^ suggest that the effect of sediment depends on spore size. Because spore size is highly variable among algal species, further studies are needed to understand the effects of sediment on different algal species.

A study on substrate adhesion in *Ulva prolifera* spores under sediment deposition showed that particles with a comparable or smaller diameter than the spores do not leave sufficient inter-particle space for spores to reach and adhere to the substrate^46^. A similar mechanism may be at play in *E. bicyclis.* That is, when small sediment particles were deposited on the substrate, the amount of free space on was reduced, and spore adhesion decreased dramatically.

For a given quantity of sediment, smaller particles had greater inhibitory effects on gametophyte growth and survival. In this experiment, we observed that many of the 15.0- and 48.2-μm particles adhered to the surface of the gametophytes. Larger numbers of fine particles on the gametophyte surface reduced the area in contact between the seawater and the gametophyte cell, obstructing nutrient uptake and gas exchange, leading to slow growth and mortality.

Gorgula & Connell^24^ suggested that although increasing sediments on rocky reefs inhibits canopy-forming macrophytes, turf algae benefit. Other studies on turf algae^23^,^25^ on rocky reefs have shown that the species are tolerant of sediment deposition, and exhibit a strong negative influence of large sediment particles on turf algae. In this study smaller sediment particles had larger negative effects on early stage canopy-forming algae. That is, sediments loads on reefs as a result of storms or floods, inhibited canopy-forming macrophyte adhesion to the substrate and slowed growth. The effects are worst from fine particle sediments, which causes the complete destruction the macrophyte community. When the canopy-forming macrophytes die, sufficient light reaches the turf-forming algae providing them with an advantageous environment. Both communities are affected by sediment in their environment.

Airoldi^31^ reviewed the influence of sediment loads on rocky reef seaweed communities in detail. Sudden or continuous sediment influx into coastal ecosystems occurring by either natural (e.g., flooding or storms) or human causes increases sediment accumulation, which has negative effects on the kelp community. There are many studies on the negative effects on the kelp communities; however, a few examples were reported in a study on the mathematical estimation of the effects of sediment^22^,^23^,^28^,^32^,^48^,^49^. Furthermore, we do not know if they estimated the effect of particle size for the influence of the sediments.

We developed an equation to estimate the early depletion of the canopy-forming macrophyte *E. bicyclis* from the particle size and quantity of sediment settling on the substrate. The equation was parameterized by exponential approximation of the experimental results performed using 15–600-μm diameter particles. The resulting equation provides a good estimate of the influence of sediment accumulation of a given particle size on the existing seaweed community. It is possible, however, that this equation may overestimate the influence of particles <15 μm. The influence of particles <15 μm requires further research.

We can evaluate the influence of sediments using equation (15) and the quantity and particle size distribution of sediments on *in situ* rocky reefs. Sudden sediment loads occur on rocky reefs during strong storms and floods. Arakawa *et al.*^50^ investigated the amount of sediments on a rocky reef seaweed forest after a river flood in Wakayama Prefecture, Japan. Using the sediment data (sediment quantity and particle size) from equation (15), the initial depletion of *E. bicyclis* in this area can be estimated.

However, we can examine the model predictions using a case study on the effects of particle transport on the coastal seaweed community off east Japan after the tsunami in the spring of 2011. It has been reported that the kelp forest in this area did not suffer significant impacts from the strong flow of the tsunami^51^. A large quantity of sediment particles rolled up by the tsunami were later gradually deposited on surviving kelp forest substrates^52^.

The tsunami that occurred in Japan in the spring of 2011 suspended large quantities of sediment particles in coastal waters that were eventually deposited on the substrate in kelp forests, among other places. *E. bicyclis* zoospores are released in autumn; thus, the tsunami occurred during the sporophyte growing season. Although the influx of particulate matter is likely to have affected sporophyte growth and survival, this effect cannot be determined from the results of the present study. Sediment influx to the rocky reef at Shizugawa in Miyagi Prefecture remained on the reef until the following year (Agatsuma *et al.*, unpublished data). Because the sediment load was up to several cm thick, zoospore adhesion is unlikely. Future monitoring of sediment loads and particle size distributions in this area will allow us to estimate the initial depletion of zoospores and gametophytes and the regrowth potential of the kelp forests.

Kelp forest destruction by coastal development, dredging and river inflow has been documented in numerous coastal areas^53^,^54^. The equations derived in this study to estimate initial kelp depletion rates could be used to evaluate the resulting negative effects on canopy-forming macrophytes in coastal ecosystems. However, we cannot understand how initial kelp depletion affects the development of future seaweed communities. We have to clarify the threshold of the initial depletion for natural kelp community formation in future study.

## Materials and Methods

The effects of sediment particles on zoospore substrate adhesion and on the growth and survival of the canopy-forming kelp *E. bicyclis* gametophytes were examined using laboratory experiments. Our experiments utilized various sediment particle sizes to the maximize relevance to actual kelp forest ecosystems. Effects on both male and female mature gametophytes were analysed. A model estimating the depletion of early stage *E*. *bicyclis* under deposition of sediment particles in the field was formulated using the laboratory results.

### Seaweed samples and sediment particles

Mature *E. bicyclis* sporophytes were collected from a subtidal reef in Shirahama, Minamiboso city, Chiba Prefecture, Japan. Sori were immediately cut from the thalli, washed, wiped dry and left in the shade for 1–2 h. A zoospore suspension was prepared by soaking the sori in sterile filtered seawater (Millipore Filter HA, 0.45 μm pore size; sterilized at 120 °C for 35 min) for 20 min to release the zoospores and then allowing the mixture to stand in the dark for 1 h.

**Figure 4:**
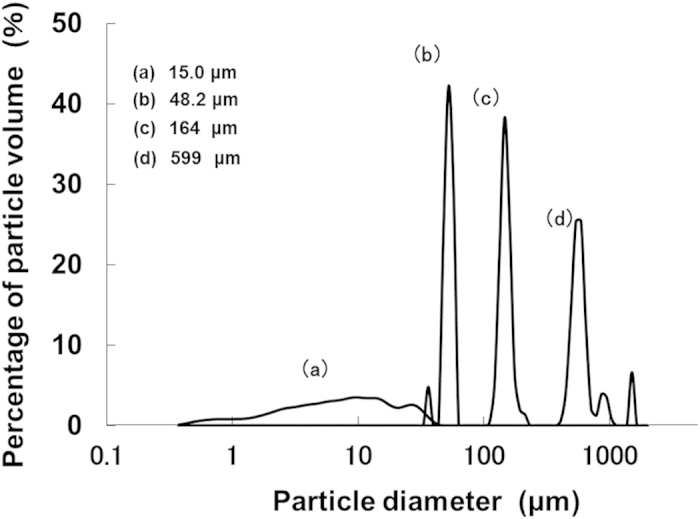
Size distribution of sediment particles.

Particles of the clay mineral kaolinite (mean diameter: 15.0 μm) and glass beads of three different diameters (mean diameters: 48.2, 164 and 599 μm) were used as the four sediment treatments (Fig. 4a–d). Particle size distributions were measured using a LS-200 instrument (Beckman-Coulter Inc., California).

### Effect of sediment particles on zoospore substrate adhesion

To determine the effect of sediment loads on zoospore adhesion to the substrate, cylindrical tanks (34 cm × 30 cm, diameter × height) were filled to a 28-cm depth with filtered seawater, and glass slides with a covering of sediment particles were placed on the bottom of the tank. Sediment treatments applied to the glass slides included: 0.5, 1, 2, 3 and 5 mg cm^−2^ of 15.0-μm particles; 3, 5, 10, 25 and 50 mg cm^−2^ of 48.2-μm particles; 5, 10, 25, 50 and 100 mg cm^−2^ of 164-μm particles; and 10, 25, 50, 100 and 150 mg cm^−2^ of 599-μm particles. Control slides had no sediment. We added 75-mL of zoospore suspension (400,000 inds. mL^−1^) to the centre of the tank and allowed it to stand for 12 h. The slides were then taken from the tank and rinsed gently with filtered seawater to remove sediment. The rate of zoospore adhesion to the slides was determined using a microscope. Five replicate samples were used for the 15.0-μm particle treatments, and 10 replicates were used for the other particle treatments. The adhesion rate was calculated from the number of adhering zoospores according to the following equation:


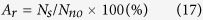


where, *A*_*r*_*, N*_*s*_ and *N*_*no*_ denote the adhesion rate and the adhesion densities in the presence and absence of sediment particles, respectively.

### Effects of sediment particles on gametophyte growth and survival

Zoospore adhesion to glass slides was accomplished by allowing a zoospore suspension of 500,000 zoospores to stand for 1 h in a tall Petri dish (15 cm × 9 cm, diameter × height) containing glass slides and 1 L sterile filtered seawater. Sediment particles were then deposited on the slides according to the following treatments: 1, 5, 10, 15 and 25 mg cm^−2^ of 15.0-μm particles; 10, 25, 50 and 100 mg cm^−2^ of 48.2-μm particles; 10, 25, 50, 100 and 150 mg cm^−2^ of 164-μm particles; and 25, 50, 150 and 200 mg cm^−2^ of 599-μm particles. Three replicates were used for each combination of particle diameter and quantity.

Seeded glass slides with sediment particles were placed two each in Petri dishes filled with PESI culture medium^55^. Petri dishes were kept in a 20 °C incubator for 12 days under neutral white fluorescent light with an intensity of 71 μmol photons m^−2^ s^−1^ and a 12: 12-h light: dark photoperiod. Six and twelve days after the start of the experiment, one slide was removed from each Petri dish. Using a microscope, the total lengths of 30–40 randomly selected gametophytes were measured, and the surviving gametophytes were counted. When the surviving gametophytes numbered less than 30–40, all live gametophytes were measured. On day 12, the body lengths of male and female gametophytes were measured separately. Discoloured gametophytes or those that had fallen off the slide were considered dead. The rate of gametophyte survival was calculated as:


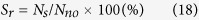


where, *S*_*r*_, *N*_*s*_ and *N*_*no*_ denote the survival rate and the densities of gametophytes after 12 days on slides with or without sediment particles, respectively.

The influence of sediment quantity and particle size on the growth of *E. bicyclis* gametophytes was analysed using ANCOVA. The terms of the model were gametophyte sex (categorical factor), quantity and size of sediment particles (covariates) and their interactions.

## Additional Information

**How to cite this article**: Watanabe, H. *et al.* Effects of sediment influx on the settlement and survival of canopy-forming macrophytes. *Sci. Rep.* **6**, 18677; doi: 10.1038/srep18677 (2016).

## Figures and Tables

**Table 1:** Gametophyte body length at 12 days under each sediment particle treatment.

Particle diameter (μm)	Sediments amounts (mg • cm^−2^)	Male			Female		
		n	Mean (%)	S.D.	n	Mean (%)	S.D.
15.0	0	61	100	15.7	59	100	14.4
	1	59	96.4	15.5	59	91.3	15.2
	5	50	92.9	15.7	50	89.2	16.2
	10	35	88.4	11.4	36	86.9	14.4
	15	33	87.3	14.3	30	88.4	17.0
48.2	0	97	100	18.2	91	100	18.0
	10	93	96.4	19.9	93	91.4	17.3
	25	93	92.2	18.1	93	86.6	16.5
	50	92	89.0	19.3	93	84.0	17.7
	100	45	84.8	17.4	45	78.5	19.7
164	0	103	100	17.6	92	100	16.1
	10	93	95.4	15.6	92	92.2	19.5
	25	63	89.5	16.2	62	88.2	19.4
	50	92	93.8	17.7	92	89.3	21.8
	100	75	90.6	16.8	78	87.5	19.2
	150	65	91.1	19.4	65	90.5	20.5
599	0	61	100	14.7	61	100	15.0
	25	61	96.0	14.6	61	96.7	15.1
	50	61	95.7	17.4	61	93.2	16.0
	100	61	88.8	17.7	61	87.4	18.5
	200	62	91.9	20.9	62	87.6	16.6

**Table 2:** ANCOVA results on the effects of sediment quantity and particle size on male and female gametophyte growth.

Source	df	MS	*F*-value	*P*-value
Gametophyte length				
Particle size	1	14769	18.84	<0.001
Amount of sediments	1	44479	56.75	<0.001
Sex	1	163419	208.49	<0.001
Particle size × Sex	1	12	0.01	0.900
Amount of sediment × Sex	1	940	1.20	0.274
